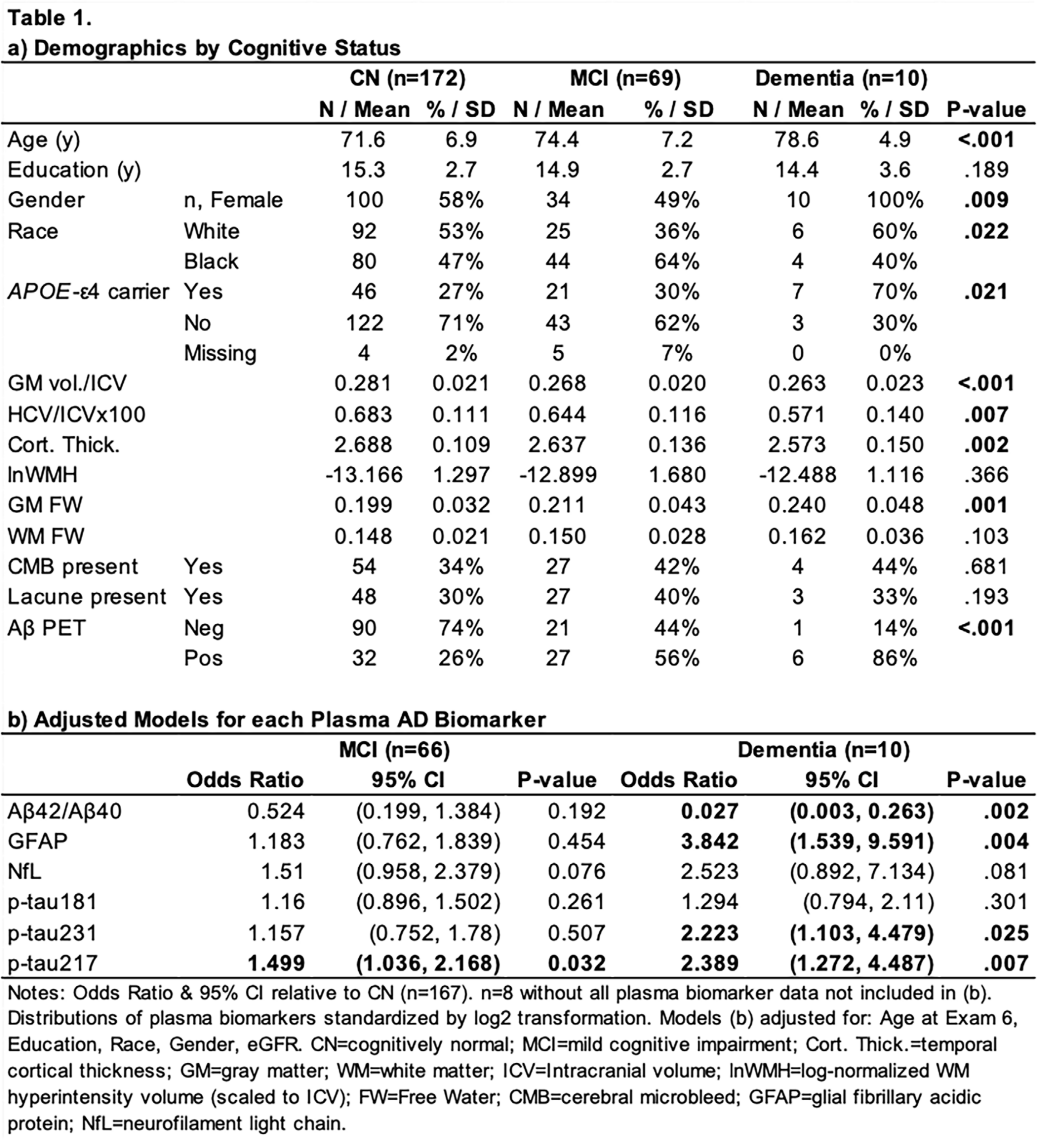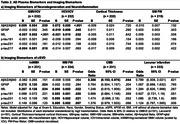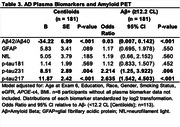# Plasma and neuroimaging biomarkers of small vessel disease and Alzheimer’s disease in a diverse cohort

**DOI:** 10.1002/alz.093879

**Published:** 2025-01-09

**Authors:** Samuel N. Lockhart, Courtney L. Sutphen, Jordan E. Tanley, Fernando Gonzalez‐Ortiz, Przemyslaw Radoslaw Kac, Mohamad Habes, Susan R. Heckbert, Nicholas J. Ashton, Michelle M. Mielke, Christopher T Whitlow, Kevin Hiatt, Suzanne Craft, Thomas C. Register, Kathleen M. Hayden, Stephen R. Rapp, Henrik Zetterberg, Kaj Blennow, Thomas K Karikari, Timothy M. Hughes

**Affiliations:** ^1^ Wake Forest University School of Medicine, Winston‐Salem, NC USA; ^2^ University of Gothenburg, Mölndal Sweden; ^3^ Glenn Biggs Institute for Alzheimer’s & Neurodegenerative Diseases, University of Texas Health Sciences Center at San Antonio, San Antonio, TX USA; ^4^ University of Washington, Seattle, WA USA; ^5^ Institute of Neuroscience and Physiology, University of Gothenburg, Mölndal Sweden; ^6^ Wake Forest University School of Medicine, Winston Salem, NC USA; ^7^ University of Gothenburg, Mölndal, Gothenburg Sweden

## Abstract

**Background:**

Little is known about how plasma Alzheimer’s disease (AD) biomarkers relate to neuroimaging biomarkers of cerebral small vessel disease (cSVD) in the context of neurodegeneration and AD pathology in late life.

**Method:**

This cross‐sectional study included 251 Multi‐Ethnic Study of Atherosclerosis (MESA) Exam 6 participants with plasma AD biomarkers (Aß42/Aß40, GFAP, NfL, p‐tau181, p‐tau217, p‐tau231; Quanterix SIMOA), MRI (neurodegeneration and cSVD), PiB (amyloid) PET, and UDSv3‐based adjudicated cognitive status (69% cognitively normal, 27% MCI, 4% probable dementia) data at the Wake Forest site. Multivariable models examined relationships among cognitive status, plasma, and neuroimaging biomarkers (covariates: age, education, race, gender, smoking status, kidney function [eGFR], APOE‐e4, BMI; significance at p<.05 uncorrected).

**Result:**

Cognitive status associated with imaging biomarkers and with plasma biomarkers of Aß42/Aß40, GFAP, p‐tau217 and p‐tau231. No NfL or p‐tau181 group differences were observed (Table 1). Lower plasma Aß42/Aß40 (Table 2) was associated with lower gray matter (GM) volume (GMV) and hippocampal volume (HCV); higher GFAP levels were associated with lower HCV. Higher NfL levels were associated with lower GMV and HCV, and with higher GM Free Water (FW). Higher p‐tau217 levels were associated with lower GMV. Plasma p‐tau231 was not associated with neurodegeneration imaging biomarkers. Higher NfL levels were associated with higher white matter (WM) hyperintensity (WMH) volume and WM FW. All plasma tau measurements were positively associated with WM FW but not other cSVD biomarkers. Lower Aß42/Aß40 ratio was significantly associated with greater prevalence of microbleeds. Higher GFAP was associated with higher WMH volume. As anticipated, lower plasma Aß42/Aß40 ratio and higher p‐tau217 and p‐tau231 were associated with higher Aß deposition and odds of Aß positivity (Table 3); a doubling in the level of p‐tau217 or p‐tau231 was associated with a 2‐3 fold increase in the odds of Aß PET positivity.

**Conclusion:**

We found that plasma GFAP, NfL, p‐tau181, p‐tau217, and to a lesser extent p‐tau231 are associated with cognitive status, vascular comorbidities and imaging biomarkers of cSVD, in addition to measures of AD‐related pathology. Importantly, we demonstrated novel associations between plasma AD biomarkers of p‐tau and NfL with cSVD measures of WM structural health.